# Case Report: Remdesivir and Convalescent Plasma in a Newly Acute B Lymphoblastic Leukemia Diagnosis With Concomitant Sars-CoV-2 Infection

**DOI:** 10.3389/fped.2021.712603

**Published:** 2021-08-02

**Authors:** Giovanni Battista Dell'Isola, Matteo Felicioni, Luigi Ferraro, Ilaria Capolsini, Carla Cerri, Grazia Gurdo, Elena Mastrodicasa, Maria Speranza Massei, Katia Perruccio, Mariangela Brogna, Alessandra Mercuri, Barbara Luciani Pasqua, Paolo Gorello, Maurizio Caniglia, Alberto Verrotti, Francesco Arcioni

**Affiliations:** ^1^Pediatric Clinic, Department of Surgical and Biomedical Sciences, University of Perugia, Perugia, Italy; ^2^Pediatric Onco-Hematology With Bone Marrow Transplantation Unit, Azienda Ospedaliera di Perugia, Perugia, Italy; ^3^Clinic of Infectious Diseases, Department of Medicine, Azienda Ospedaliera di Perugia, University of Perugia, Perugia, Italy; ^4^Immunohaematology, Transfusion Service and Apheresis, Azienda Ospedaliera di Perugia, Perugia, Italy; ^5^Department of Chemistry, Biology and Biotechnology, University of Perugia, Perugia, Italy

**Keywords:** leukemia, COVID-19, remdesevir, SARS–CoV−2, convalescent plasma

## Abstract

**Introduction:** The spread of Covid-19 has worsened the prognosis of oncology patients, interrupting or delaying life-saving therapies and contextually increasing the risk of severe SARS-CoV-2 infections. Acute lymphoblastic leukemia (ALL) is the most frequent cancer in pediatric age and the management of this disease with concomitant SARS-COV-2 infection represents a challenging situation.

**Case presentation:** We present the case of a 6-year-old female newly diagnosed with ALL during a documented SARS-CoV-2 infection. Our patient was admitted 20 days after SARS-CoV-2 detection for evening-rise fever. Laboratory testing showed severe neutropenia while chest x-ray detected moderate pulmonary involvement. Acute lymphoblastic leukemia diagnosis was made through morphological and molecular analysis on bone marrow aspirate. Given the stability of the blood count and clinical conditions, antiviral therapy with Remdesivir and Convalescent Plasma was started before antileukemic treatment, obtaining a rapid resolution of the infection.

**Conclusion:** In our experience, the treatment with Remdesivir and Convalescent Plasma led to a rapid resolution of Sars-Cov-2 infection. Our case did not present any adverse event to the therapy. Thus, this treatment could be considered in patients with malignancies, in order to accelerate the resolution of the infection and begin immunosuppressive treatment safely. Further studies are required to confirm this hypothesis.

## Introduction

Acute lymphoblastic leukemia (ALL) is the most frequent cancer in pediatric age with an incidence peak between 2 and 7 years of age. B-ALL is the most common subtype, accounting for ~80% of ALLs. The interaction between polymorphic variants in several genes and environmental factors, such as infections, is a recognized cause of ALL. Based on Greaves model, Covid-19 has been proposed as a “second hit” in the genesis of ALL (1).

During the pandemic, SARS-CoV-2 infections have been described in patients with malignancies (2) as well as severe Covid-19 diseases in pediatric oncology patients (3). Nevertheless, the immunosuppression influence on the infection course is not well-known and its predisposition to a worsen prognosis is still under discussion (4). As far as the management of pediatric oncology patients is concerned, it could be prudent to defer high-intensity treatments when possible (5).

Only a few cases presented ALL onset with concomitant Covid-19, and it is still debated whether to start chemotherapy before or after Covid-19 resolution. While some authors reported their experience without chemotherapy delay nor Covid-19 treatment (6), others postponed chemotherapy or associated it with antiviral therapy (7–9).

Covid-19 treatment in pediatric age is limited by the absence of specific indications. Remdesivir has been suggested for children with severe or critical illness (10). There are few reports of the use of Convalescent plasma (CP) in the pediatric population (11). Baricitinib used in combination with Remdesivir, resulted effective in reducing hospitalization in patients with Covid-19 (12). There is data that advise against the use of monoclonal antibodies (bamlanivimab or casirivimab and imdevimab) in children and adolescents with COVID-19 (13). Bamlanivimab binds to the receptor binding domain (RBD) of the SARS-CoV-2 spike protein. Casirivimab and imdevimab bind to non-overlapping regions of the SARS-CoV-2 RBD. The use of these monoclonal antibodies is authorized from 12 years of age in patients not requiring hospitalization at high risk for progressing to severe COVID-19 and/or hospitalization. However, neither bamlanivimab nor casirivimab plus imdevimab should be considered safe and effective in pediatric population (13).

Here, we present the case of a 6-year-old female who was newly diagnosed with ALL during a documented SARS-CoV-2 infection. The aim of this work is to share and to discuss our approach to this challenging situation in the absence of common guidelines.

## Case Presentation

**Figure 1:**
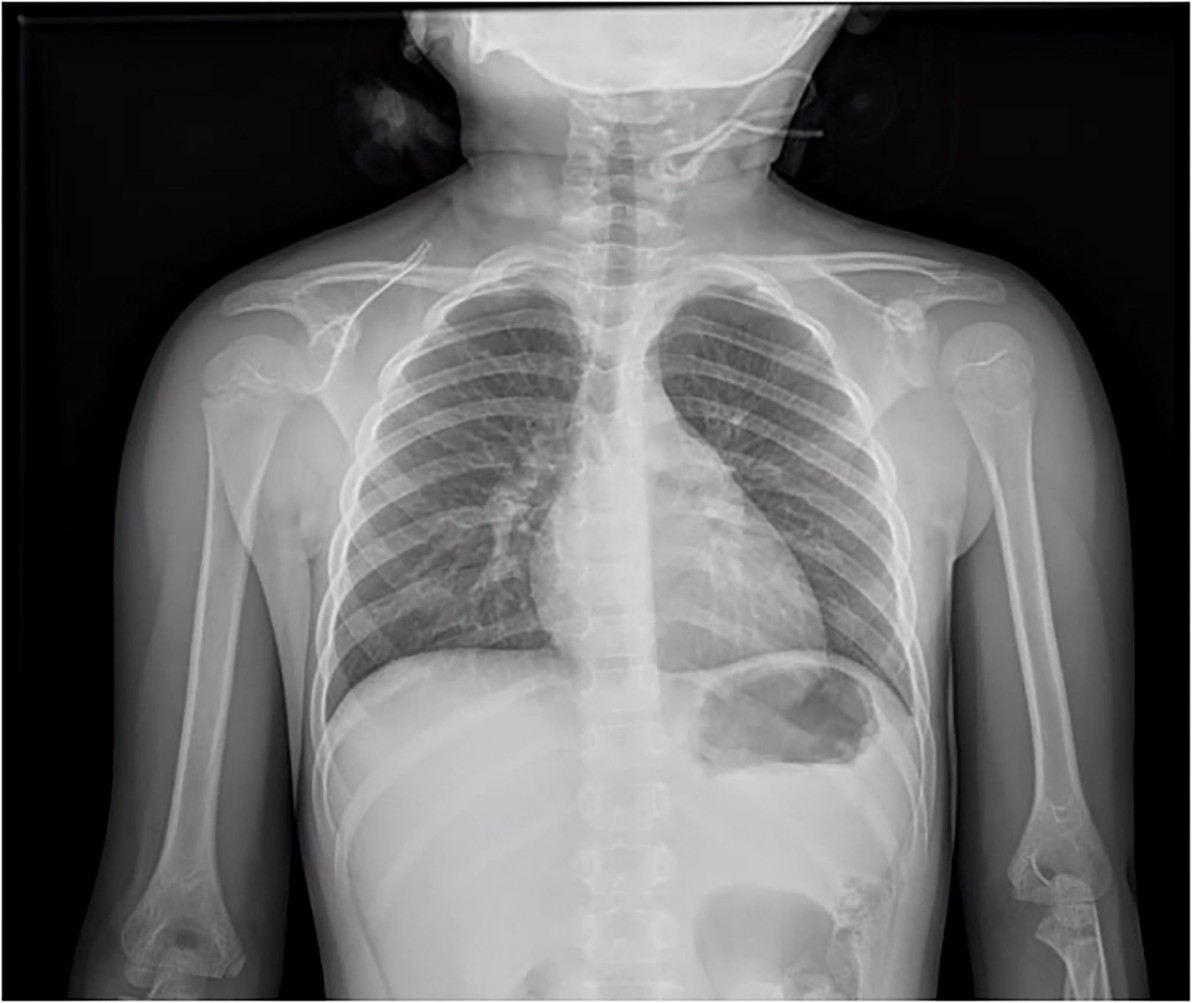
Chest x-ray showed slight thickening of the interstitial web with mild bilateral pleural effusion.

**Figure 2:**
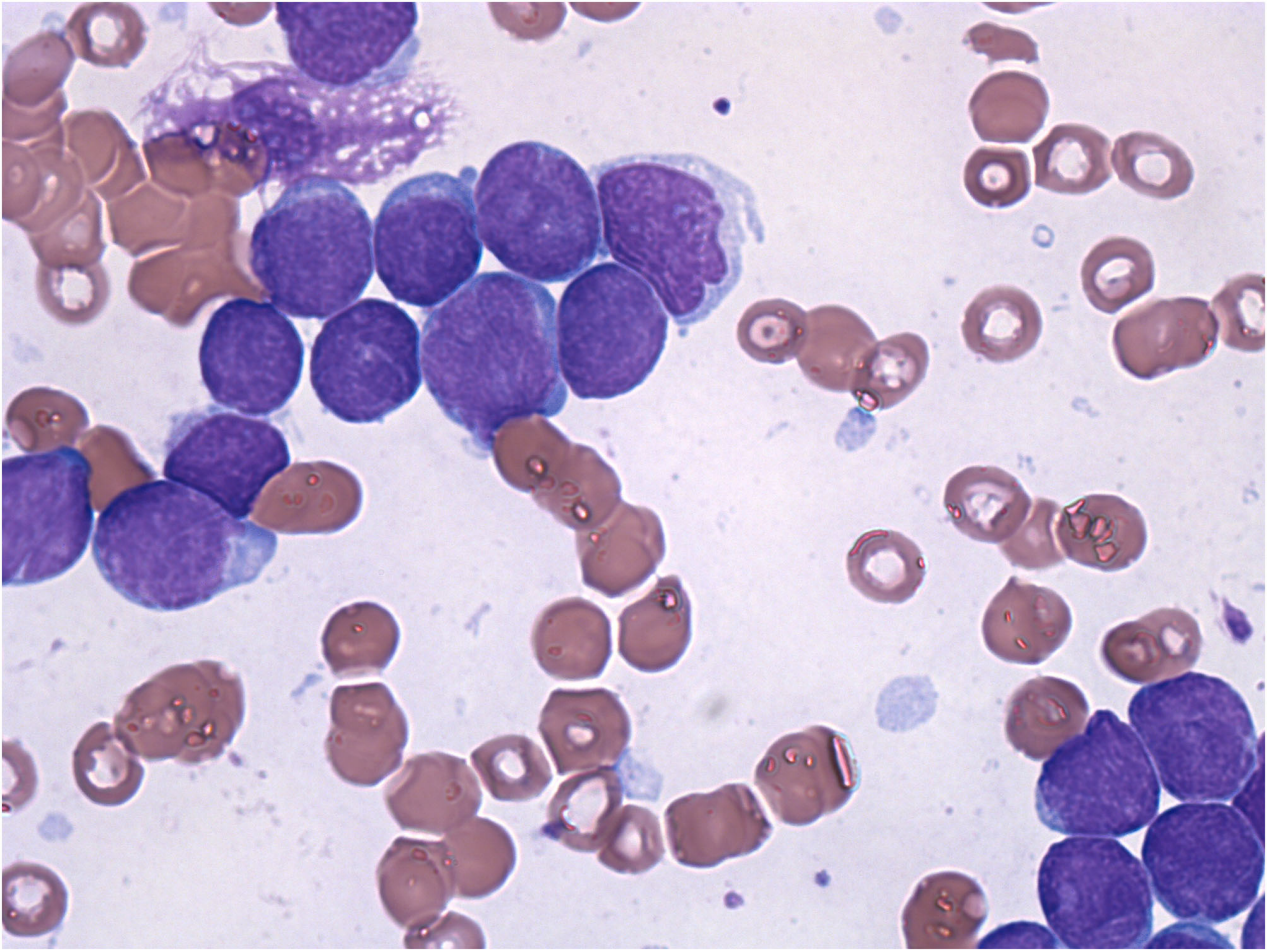
Morphological analysis of the bone marrow aspirate shows rich cellularity, more than 90% of which is replaced by blastic elements with a probably lymphoid habitus and a high nucleus/cytoplasm ratio.

**Figure 3:**
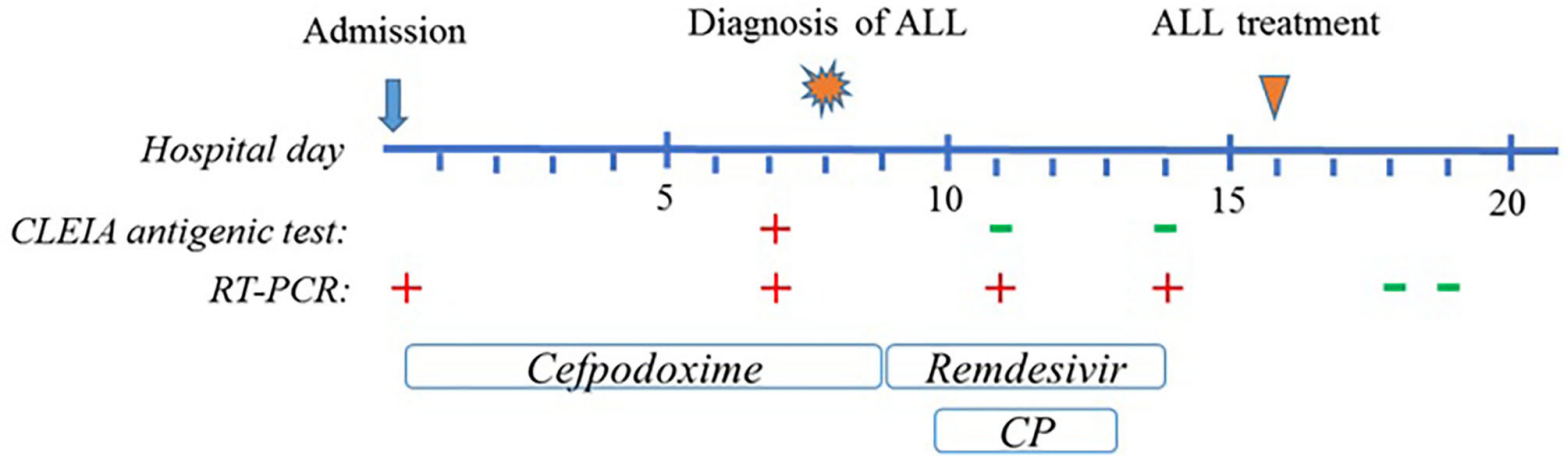
Graphical representation of clinical course of the patient. Timeline includes up to 20 days after admission, until the SARS-CoV-2 molecular test resulted negative.

A 6-year-old girl was admitted to the department of Pediatrics for intermittent evening-rise fever in the previous three months during Sars-Cov-2 infection that was detected 20 days before the admission through a PCR test. The frequency of febrile episodes was one per week. At first examination, she presented no clinical signs of infection and no needs of oxygen supplementation. Blood laboratory tests showed: white blood cells (WBC) 3460/mmc, neutrophils 218/mmc, lymphocytes 2951/mmc, monocytes 8.1%, eosinophils 0%, basophils 0.3% hemoglobin 11 g/dL, platelets 242000/mmc, LDH 487 UI/L (normal values 0–247), uric acid 4.5 mg/dL, along with C-reactive protein 5.6mg/dL and interleukin-6 9.5 pg/ml. Other parameters, including transaminases, blood urea nitrogen, creatinine, prothrombin time and activated partial thromboplastin time were normal. SARS-CoV-2 infection was confirmed by rapid test Xpert Xpress SARS-CoV-2 (Cepheid) and quantitative antigenic tests. It was not possible to sequence viral genome. Chest x-ray showed slight thickening of the interstitial web with mild bilateral pleural effusion (Figure 1). Abdominal ultrasound scan was normal except for minimal perisplenic and perihepatic fluid layers. Through PCR and serological tests, we excluded an acute infection caused by HIV, HCV, HBV, CMV, Parvovirus, Enteric virus, Salmonella, Shigella, Yersinia, and Brucella. Antibiotic treatment with Cefpodoxime was started for presumed pneumonia and continued for a total of 9 days without resolution of the fever. Severe neutropenia persisted at follow-up. Peripheral blood smear was examined with evidence of lymphoid elements, with a high nucleus-cytoplasm ratio and dispersed chromatin, of uncertain interpretation. Immunophenotype on peripheral blood showed signs of immunoproliferative disorder. The morphological and molecular analysis on bone marrow aspirate confirmed the presence of blasts (76%) CD45+, CD19+, CD10+, CD22 –/+ (38%), MPO–, cCD79a+ diagnostic for B cell acute lymphoblastic leukemia (Figure 2). The cytogenetic analysis showed a hyperdiploid karyotype, with trisomic hybridization pattern for the CRFL2, CDKN2A, ABL1, IGH genes, and tetrasomy of RUNX1. Bone marrow biopsy was performed showing diffuse infiltration of blasts. CSF analysis showed no central nervous system involvement (1 WBC/UL). SARS-CoV-2 RT-PCR tests were persistently positive. Thus, given the stability of the blood count and clinical conditions, antiviral therapy was started with Remdesivir initially at 5 mg/kg intravenously, followed by 2.5 mg/kg daily for a total of 5 days and 10 ml/kg of CP for 3 days. The CP administered was against the original strain of the virus with an antibody titer of 1:20. However, the antibody neutralization test on the Brazilian variant P.1 resulted positive (IgG titer 1:160) at a later stage. Hepatic function monitoring showed no increase of transaminases. No hypersensitivity reactions were described. Chemiluminescence enzyme immunoassay (CLEIA) for SARS-CoV-2 antigen resulted negative 2 days after the first Remdesivir administration while PCR tests were still positive. At the end of antiviral therapy, the patient condition was deteriorating with progressive anemia. Thus, prednisone therapy was started according to AIEOP-BFM ALL 2017 protocol (14). Two days later, also the SARS-CoV-2 RT-PCR test resulted negative (Figure 3). The peripheral blood smear examination on day +8 showed no blast cells, the bone marrow examination on day +15 and on day +33 was compatible with complete hematological remission.

## Discussion

We report the case of ALL diagnosed during a SARS-CoV-2 infection. Although SARS-CoV2 has rarely caused severe forms in children, immunocompromised patients with malignancies may be at higher risk for severe disease (15). ALL onset with concomitant Covid-19 represents one of these challenging situations and its management is debated and currently based on clinician experience and the patient condition. Covid-19 course during immunosuppressive therapy is still uncertain and may differ from that of the common population. Covid-19 is characterized by a hyperimmune reaction in the early stages of the disease. Thus, immunocompromised patients could present a less severe infection, with a slow rate of negativization. However, lung damage such as that present in our case can worsen in the most advanced stages of infection, probably due to direct damage by persistent viral replication caused by immunological deficiency (16).

Although rarely, compared to the adult population, severe pediatric cases of Covid-19 required admission to intensive care unit and were associated with adverse clinical outcome (17). There are no official guidelines defining the treatment of Covid-19 in the pediatric population. Remdesivir should be considered for severe and critical disease and has been used in immunocompromised patients. However, there is limited evidence to define immunodeficiency as a risk for severe forms of Covid-19 and each situation should be evaluated on a case-by-case basis (10). CP has been proposed as a therapy to reduce the severity and shorten the duration of the disease especially at high antibody titer (18). According to adult population studies, except for patients treated in the very early stages of the disease, CP did not show efficacy in reducing mortality and the need for invasive ventilation in moderate-to-severe disease. Moreover, there is evidence of significant loss of CP neutralizing activity against Gamma Variant of Concern (VOC) formerly known as P.1. However, although the results were not statistically significant, a recent Italian study supported a potential therapeutic role of CP in subjects with mild-to-moderate Covid-19. To date, only 6 studies have described the use of CP in the pediatric population with severe Covid-19 and/or immunodeficiency with concomitant SARS-CoV-2 infection. All resulted in good outcomes and no adverse events (11, 16, 19–22).

In our case CP was administered before knowing which strain the neutralization test was positive for, in order to accelerate the start of treatment for Acute Leukemia. (23–25). At the time of the patient's infection, the epidemiology of Sars-Cov-2 infection in Umbria (identified as red zone) showed about 20% of infections deriving from the Gamma VOC and almost all the other cases from Alpha VOC. However, almost all of the previously collected hyperimmune plasma was against the standard virus strain. Our approach was to give the CP to the patient despite the epidemiological data reported above, considering the risk-benefit ratio favorable. Indeed, the administration of plasma in a protected environment is a relatively safe procedure. In order to enhance the efficacy of the treatment, the plasma was administered as soon as practicable, even though our patient was likely in a late phase of the infection.

It is certainly very difficult to establish with certainty whether it was the infection with Sars-Cov-2 or the leukemic stem cells that originated first. The positivity of RT-PCR tests for Sars-Cov-2 infection dates back to about 20 days before the diagnosis of ALL. On the other hand, the patient's blood count remained stable for a long time in the treatment phase with remdesivir and hyperimmune plasma. It is therefore likely that the two pathological occurrences had a similar temporal origin. Further data and studies are needed to be able to hypothesize a univocal or bi-univocal relationship between the two pathologies, which at the moment is not possible to assume. Our patient was in good clinical condition with a stable blood count at the diagnosis of ALL. We decided to postpone chemotherapy in an attempt to speed up healing from SARS-CoV-2 infection. Remdesivir was started in compassionate use according to the FDA recommended dosage (26). In order to quickly start chemotherapy, CP was added to the antiviral. The patient's deteriorating condition led us to start the induction therapy before the RT-PCR test was negative. However, when steroid therapy was started CLEIA antigen test was already negative with positive neutralizing antibodies and RT-PCR test resulted negative 2 days after starting immunosuppressive therapy. To corroborate this hypothesis, no sign of severe Covid-19 was detected in our patient afterwards.

In conclusion, the double therapy with remdesivir and CP may have led to a rapid resolution of the infection within 8 days, meant as negative RT-PCR tests. No adverse events were described. Limitation of this study is the uncertainty in defining active SARS-CoV-2 infection in our patient. The child had fever but no other symptoms of Covid-19 except for non-specific pulmonary involvement and neutralizing antibodies titer already positive on admission. Therefore, it is questioned whether the patient was already recovered from Covid-19 prior to remdesivir and CP therapy. In fact, in the late phase of the disease, RT-PCR tests may show false positive results due to prolonged viral RNA shedding detected at high cycle threshold values (27, 28). Analysis of inflammation indices, as markers of infection activity, showed high d-dimer (824 ng/ml n.v. 0–500) and normal ferritin (22.6 ng/ml v.n. 11–307). This could lead to the iteration of a prolonged positivity of the immunocompromised patient in the absence of active infection. However, it is also known that an immunocompromised patient has a functional anergia that could lead to lower levels of inflammation compared to immunocompetent host. In addition, it is worth notice that CLEIA test resulted positive after admission suggesting ongoing infection. On the basis of a single case it is not possible to draw definitive conclusions, however we believe that it is useful to initiate antiviral therapy in order to accelerate healing from SARS-CoV-2 infection. According to our experience, this treatment could be considered in patients at high risk of severe Covid-19 with underlying malignancies. Nevertheless, antileukemic therapy cannot be delayed especially with clinical-laboratory signs of disease progression.

To the best of our knowledge, this is the first case report describing the treatment of a pediatric patient with ALL onset and Covid-19 with Remdesivir and CP. Although this is a single case, we hope with this work to give a contribution in the management of this challenging situation and to encourage the publication of more case reports in order to develop common guidelines.

## Data Availability Statement

The original contributions generated for the study are included in the article/supplementary material, further inquiries can be directed to the corresponding author/s.

## Ethics Statement

Ethical review and approval was not required for the study on human participants in accordance with the local legislation and institutional requirements. Written informed consent to participate in this study was provided by the participants' legal guardian/next of kin. Written informed consent was obtained from the parents/guardians of the patient for publication of this case report and any accompanying images. A copy of the written consent is available for review by the Editor of this journal.

## Author Contributions

GD, MF, and FA conceived the work, collected the data, written, and reviewed the work. AV and MC extensively revised the work. PG, IC, CC, GG, KP, EM, MB, MM, LF, and AM took care of the clinical management of the patient. BP dealt with the management of hyperimmune plasma against COVID 19. All authors have read and approved the manuscript.

## Conflict of Interest

The authors declare that the research was conducted in the absence of any commercial or financial relationships that could be construed as a potential conflict of interest.

## Publisher's Note

All claims expressed in this article are solely those of the authors and do not necessarily represent those of their affiliated organizations, or those of the publisher, the editors and the reviewers. Any product that may be evaluated in this article, or claim that may be made by its manufacturer, is not guaranteed or endorsed by the publisher.

## Abbreviations

ALL: Acute lymphoblastic leukemia
CP: Convalescent plasma.
